# *Echinops* as a Source of Bioactive Compounds—A Systematic Review

**DOI:** 10.3390/ph18091353

**Published:** 2025-09-09

**Authors:** Simona Ivanova, Alexandra Ivanova, Mina Todorova, Vera Gledacheva, Stoyanka Nikolova

**Affiliations:** 1 Department of Organic Chemistry, Faculty of Chemistry, University of Plovdiv, 4000 Plovdiv, Bulgaria; simonaivanova1108@gmail.com (S.I.); ivanova.aleksandra@uni-plovdiv.bg (A.I.); minatodorova@uni-plovdiv.bg (M.T.); 2 Department of Medical Physics and Biophysics, Faculty of Pharmacy, Medical University of Plovdiv, 4002 Plovdiv, Bulgaria; vera.gledacheva@mu-plovdiv.bg

**Keywords:** *Echinops*, *Asteraceae*, thiophenes, alkaloids, anti-Alzheimer, antidiabetic, antimalarial, antibacterial, insecticidal

## Abstract

**Background**: *Echinops* is a genus of spiny, herbaceous perennials in the *Asteraceae* family, known for its distinct morphology and broad pharmacological potential. Both traditional and modern medicinal systems have identified species in this genus as sources of bioactive compounds with anti-inflammatory, antimalarial, antidiabetic, anticancer, and neuroprotective effects. **Aims**: This study aimed to conduct a systematic literature review and update previous overviews of the recently reported phytochemicals and pharmacological properties of *Echinops*, systematically summarizing biological activities and their therapeutic applications. **Methods**: Major electronic medical databases—PubMed, Scopus, Science Direct, Web of Science, and Google Scholar—were systematically searched for publications from 1990 to 2025. **Results**: A total of 134 studies met our inclusion criteria. Thiophenes and terpenes emerged as characteristic metabolites of the genus, and along with flavonoids and alkaloids, contributed to a wide range of bioactivities. Experimental evidence supports the potential of these compounds as multifunctional agents, although clinical validation remains limited. **Conclusions**: *Echinops* is a promising source of structurally diverse metabolites with therapeutic relevance. Further pharmacological and toxicological studies are needed to establish their efficacy and ensure safe medical application.

## 1. Introduction

The genus *Echinops* L., popularly referred to as globe thistles, belongs to the *Asteraceae* family of daisies. With over 120 species, this genus is found worldwide, primarily in the northern hemisphere [1].

The presence of uniflowered capitula aggregated into second-order spherical or oval heads identifies the genus *Echinops*, which is a member of the *Cardueae* tribe [2,3]. It is distinct within the tribe because of this trait. *Echinops*’ great morphological homogeneity makes its taxonomical delimitation virtually uncontested, but it also makes it more difficult to create natural groups and infrageneric classification. The *Echinops* genus has advantages in both the medical and ecological fields. Potential anti-inflammatory, antibacterial, and analgesic properties of the bioactive chemicals derived from *Echinops* extracts have been discovered by recent pharmacological investigations. These results suggest the genus’s significance in plant-based drug development [4].

### Botanical Identity, Taxonomy, and Cultivation

**Figure 1:**
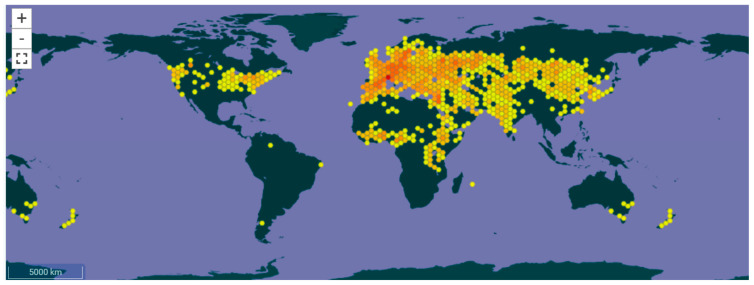
Global distribution map of *Echinops* L. Reproduced from GBIF.org (accessed on 21 August 2025), under the terms of the Creative Commons Attribution 4.0 License [5].

Based on the geographic distribution of taxa, a variety of *Echinops* species have been identified in eight geographic regions, including Eastern Europe, Western Europe, Middle Asia, East Asia, Irano-Turanian Region, North Africa, Tropical Africa, and the Arabian Peninsula (Figure 1).

Likely originating from steppe regions of Southeast Europe and West Asia, *E. sphaerocephalus* is native to temperate parts of Asia and Southern and Eastern Europe [6]. *Echinops exaltatus* Schrad., *Echinops banaticus* Rochel ex Schrad., and *Echinops ritro* L. are three other species that are found in Europe.

There are eight taxa known to exist in Italy. Currently, 12 taxa are known to exist in Greece, while nine are present in Albania [7]. Thirteen taxa are found in Azerbaijan, twelve in Armenia, six in Russia, three in Austria, the Czech Republic, Hungary, and Slovakia, and twenty in Turkey [8,9,10,11,12].

The perennial plant has straight, thorny wooden stems and grows to a height of 30 to 100 cm. The inflorescences are tubular flowers that lead to thorns and are blue and white–silver in color. The leaves are long and have serrated edges without petioles [13]. The genus’s axillary, terminal, compound, single-flowered capitula coalesce into a globose synflorescence. Every capitulum is sessile, supported by tiny, hidden bracts, and disintegrates when it reaches maturity. The capitula are made up of an inner series of imbricate, inflexible, free, or partially joined phyllaries and an outer series of simple or branching extraphyllary white bristles [14,15]. The genus’s corolla may be monomorphic, tubular, glandular, or glabrous and features five lobes that can be white to cream, yellow, pink to red, pale to deep blue, or violet. Florets are tubular and bisexual. The anthers are either violet or purplish. The stigmata are purple or white. Achenes are thickly appressed-pilose and elongated, oblong, or obovate. There are persistent, free, crown-like, or short connate scale-like bristles on the pappus. The single-seeded capitulum is the deciduous unit of dissemination.

More than 120 species of this genus can be found worldwide, particularly in Central Asia, the Mediterranean Basin, and tropical Africa [16]. It grows between 0 and 400 m (0 and 1312 feet) above sea level in areas that are sunny, rocky, or brushy and have more or less mineral-rich soils. The *Echinops* ritro-globe-thistle species is found across Bulgaria at elevations ranging from 200 to 1500 m. It grows best in rocky and grassy environments [17].

The perennial accessions of *Echinops sphaerocephalus*, *E. exaltatus*, and *E. banaticus* grow in loess soils in Halle/Saale, Germany, in an arid region (104 m above sea level, 51729′ northern latitude, 11759′ eastern longitude, average annual temperature 9.0 7C, rainfall less than 500 L/m^2^ annually).

*E. sphaerocephalus* grows best in dry, slightly alkaline soils, suggesting that the plant may be successfully cultivated in areas where other oil plants, such as rapeseed, do not produce abundant harvests. According to descriptions, achene fruits of the *Asteraceae* family are typically 4–6 mm long.

It can reach a height of 1.5 m and favors calcareous soils. The plant gets its name from the blue or white blossoms that form spherical racemes (3 to 8 cm in diameter) during the July to August blooming season (lat. *Echinops*—hedgehog, ops—looks). According to [18], seeds have a 25% oil content and ripen in early fall, between September and October. *E*. *sphaerocephalus* typically spreads from areas where it was previously artificially planted, but it does so extremely slowly [19].

For optimal development, it requires alkaline, well-drained soils. The majority of *Echinops* species can tolerate a variety of soil types, including fresh and alkaline soils.

The objective of this systematic review is to comprehensively evaluate the phytochemical constituents and pharmacological activities of *Echinops* species, and to postulate their potential therapeutic applications based on current preclinical and clinical evidence. Despite the broad pharmacological interest in the genus *Echinops*, there remains a noticeable lack of detailed phytochemical and pharmacological data. This gap is even more pronounced in the context of Bulgaria, where comprehensive studies on native *Echinops* species are scarce or fragmented.

## 2. Materials and Methods

This review aimed to critically evaluate available research on the genus *Echinops* and systematically organize and present the findings. An attempt was made to include all articles published from 1990 to 2025. Some articles published before 1990 were included based on their significance. Relevant studies were identified using databases such as PubMed, Scopus, ScienceDirect, Web of Science, and Google Scholar. Keyword combinations such as “Echinops,” “nutritional composition,” “phytochemistry,” “pharmacological activity,” “bioactive compounds,” “antioxidant,” “anti-inflammatory,” “traditional medicine,” and “functional foods” were among the search results. Two independent reviewers screened the titles and abstracts of all retrieved records to assess eligibility. Full texts of potentially relevant articles were then evaluated against the inclusion and exclusion criteria. Any disagreements were resolved through discussion or consultation with a third reviewer. No automation tools were used in the selection process. English- and German-language, peer-reviewed journal publications were included. Original research studies and review articles that discussed the pharmacological, chemical, nutritional, botanical, or commercial aspects of *Echinops* spp. were taken into consideration. When discussing pharmacological effects, studies that were based on in vitro, in vivo, or clinical evaluations were given priority. Articles that were outside of the scope of the review, duplicate entries, or non-peer-reviewed materials were not included. In order to present a systematic review of the current understanding of *Echinops* spp., with consideration for both traditional and contemporary applications, the chosen research was subjected to critical evaluation and arranged topically.

**Figure 2:**
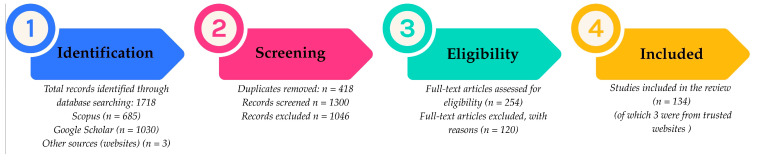
PRISMA flowchart illustrating the literature review’s identification, screening, eligibility, and inclusion process. Created with Canva (www.canva.com).

To conduct this literature search, various databases were utilized, including PubMed, Google Scholar, Springer Nature, Scopus, Medline, ScienceDirect, and Elsevier. The search employed keywords such as anti-Alzheimer, antidiabetic, anti-malarial, antimicrobial, cytotoxicity, *Echinops*, and phytochemistry (Figure 2). Research articles served as the primary sources for the structures of isolated or synthesized compounds. Our search strategy included recently published accessible data. In addition to global data, our search also included region-specific studies, particularly those focusing on *Echinops sphaerocephalus* and species native to or studied within Bulgaria. Although several *Echinops* species have been studied globally, data specifically addressing *Echinops sphaerocephalus* and the genus as represented in the Bulgarian flora remain limited. This underrepresentation may hinder a full understanding of the species’ potential within regional ethnopharmacology and biodiversity. The primary outcomes sought included pharmacological activities such as antioxidant, anti-inflammatory, antidiabetic, antimalarial, antimicrobial, cytotoxic, and neuroprotective effects. When applicable, data were extracted for all outcome measures reported (e.g., IC50 values, bacterial strains, MIC, biochemical markers, cancer cell lines, clinical efficacy outcomes). Additional data collected included species name, plant part used, geographic origin, type of extract or compound, study design (in vitro, in vivo, or clinical), dose and duration of exposure, and funding sources (when reported). When data were unclear or missing, assumptions were based on standard interpretations of the methodology sections, unless clarified by the authors. Due to the heterogeneity of the included studies, results were synthesized narratively and presented. No quantitative meta-analysis was performed.

This systematic review was conducted following the PRISMA 2020 guidelines.

## 3. Results and Discussion 

The chemical content of *Echinops* L. has been the subject of many studies. The main constituents of the genus *Echinops* are terpenes and thiophenes. Flavonoids, phenolic compounds, alkaloids, lipids, and phenylpropanoids have also been reported [20]. The majority of terpenes and flavonoids are extracted from the aerial portion of the plant, while thiophenes are found in the roots. Every morphological section of the plant is said to contain some essential oils, making the genus well known for its essential oil content. It is estimated that over 53 of the identified and isolated compounds have various biological functions. A variety of extracts, essential oils, and chemicals isolated from this genus have been demonstrated to have distinct biological effects, primarily anti-inflammatory, anti-proliferative, and anti-microbial [20].

### 3.1. Thiophenes

The primary bioactive components of the genus *Echinops*, thiophenes, are produced biosynthetically from reduced sulfur and fatty acids [21]. The thiophenes are typical constituents and occur in considerable variation. They are characterized by one or three thiophene rings in their structures (Table 1).

The majority of thiophenes have two thiophene rings in their structures, and the majority of thiophenic compounds have an acetylenic functional group. The two most prevalent thiophenes identified from nine species were α-terthiophene and 5-(but-3-en-1-ynyl)-2,2′-bithiophene. Essential oils extracted from the many plants in this genus were shown to contain thiophenes. Thiophenes have been shown to have insecticidal, anti-proliferative, and anti-fungal properties. Most of the biological activities of *Echinops* L. are due to the presence of thiophenes in their extracts.

Lam et al. used GC-MS to analyze the thiophene content in diethyl ether extracts from roots, leaves, and stems of 16 *Echinops* species. The authors identified thiophenes only in the roots. Two thiophene dimers, cardopatine and isocardopatine, were found for the first time in *E. bannaticus* and *E. ritro* [34].

Liu et al. isolated and structurally elucidated 12 thiophenes from ether and n-butanol extracts of *E. grijisii* roots [35].

The hexane fraction from the roots of *Echinops ellenbeckii* O. Hoffm. from Ethiopia was examined by Hymete et al. The fraction yielded seven acetylenic thiophenes, five of which were reported for the first time in this species. The monothiophenes 5-(penta-1,3-diynyl)-2-(but-3-en-1-ynyl)-thiophene, 5-(penta-1,3-diynyl)-2-(4-acetoxy-but-1-ynyl)-thiophene, 5-(penta-1,3-diynyl)-2-(3-hydroxy-4-acetoxybut-1-ynyl)-thiophene, 5-(penta-1,3-diynyl)-2-(3,4-diacetoxy-but-1-ynyl)-thiophene, 5-(penta-1,3-diynyl)-2-(3-chloro-4-acetoxy-but-1-ynyl)-thiophene, 5-(penta-1,3-diynyl)-2-(3,4-epoxy-but-1-ynyl)-thiophene, and the dithiophene 5-[(5-acetoxymethyl-2-thienyl)-2-(but-3-en-1-ynyl)]-thiophene was also isolated [33].

Later, Wand et al. isolated and structurally elucidated four thiophenes from a 95% ethanol extract of *E. latifolius* roots [42].

Zhang et al. found eight thiophenes, including one new one, echinothiophenegenol, in a 95% ethanol extract of roots of *Echinops grijisii* Hance (Table 1) [30].

Nakano et al. isolated two polyacetylene thiophenes, echinopsacetylenes A and B, from the roots of *Echinops transiliensis* (Table 1). Echinopsacetylenes A is the first natural product possessing an R-terthienyl moiety covalently linked with another thiophene moiety. Echinopsacetylenes B is the first natural thiophene conjugated with a fatty acid moiety [40].

Wu et al. isolated a new thiophene, 2,2-Dimethyl-4-[5-(prop-1-ynyl)-2,2-bithiophen-5-yl]-1,3-dioxolane, from ethanol extracts of *Echinops spinosissimus* subsp. *spinosus* roots [50].

Li et al. found three new substituted bithiophenes (5′-(3,4-Dihydroxybut-1-yn-1-yl)-[2,2′-bithiophene]-5-carbaldehyde, 4-Hydroxy-1-(5′-methyl-[2,2′-bithiophen]-5-yl)butan-1-one, and 5′-(3,4-Dihydroxybut-1-yn-1-yl)-[2,2′-bithiophene]-5-carboxylic acid, Table 1) in a 95% ethanol extract of the entire *Echinops ritro* plant, together with twelve known substituted thiophenes, including arctinol b, 4-(5-(penta-1,3-diyn-1-yl)thiophen-2-yl)but-3-yne-1,2-diol, [2,2′-bithiophene]-5-carboxylic acid, 4-([2,2′-bithiophen]-5-yl) but-3-yne-1,2-diol, junipic acid, arctinal, 4-(5′-methyl-[2,2′-bithiophen]-5-yl)but-3-yn-1-ol, 1-([2,2′-bithiophen]-5-yl)ethan-1-one, 4-([2,2′-bithiophen]-5-yl)but-3-yn-1-ol, 1-([2,2′-bithiophen]-5-yl)-4-hydroxybutan-1-one, arctinol A, and arctic acid. The structures were elucidated based on extensive spectroscopic analysis, including 1D, 2D NMR, and MS [37].

### 3.2. Terpenes

One of the taxa in the *Asteraceae* family with well-characterized terpenes is the genus *Echinops*. Sesqui- and triterpenoids have been isolated mainly from the whole plant and aerial parts of the genus *Echinops*. Lactones are included in the majority of sesquiterpenoids. Sesquiterpene lactones are also the most common secondary metabolites in the *Asteraceae* family [55,56]. Most triterpenoids occur in a variety of forms, including lactones, esters, sterols, and their glycosides.

The entire plant and aerial portions of the genus *Echinops* are primary sources of sesqui- and triterpenoids [27,39]. The most common sesquiterpenoid reported is costunolide, which has been isolated from *E. amplexicaulis*, *E. kebericho*, and *E. pappii* [23,24] (Table 2). Abegaz et al. examined the volatile fractions of *E. hispidus* and *E. giganteus* and found the sesquiterpene lactone caryophyllene epoxide [23,41,57,58].

Four triterpenoids, namely α-amyrin, α-amyrin acetate, β-sitosterol, and sitosteryl 3-β-glucoside, were isolated from *E. ritro* [60].

Lupeol and lupeol acetate are common triterpenoids isolated from *E. niveus* [64], *E. giganteus* [65], *E. integrifolius* [66], *E. echinatus* [67], and *E. albicaulis* [47].

Phytochemical investigation of the aerial parts of *Echinops spinosissimus* led to the isolation of nine triterpenoids, including a newly reported natural product 20-oxo-30-nortaraxast-21-en-3β-ol (Table 2) [69].

Hamdan et al. examined the *n*-hexane fraction of *Echinops taeckholmiana* Amin and identified four compounds that were isolated from the defatted root extract; these compounds were identified as taraxeryl acetate, *β*-sitosterol, and stigmasterol-3-*β*-d-glucoside (Table 2) [61].

Jin et al. later isolated one dimeric sesquiterpene, Latifolanone A, and Atractylenolide (Table 2) in the CH_2_Cl_2_-soluble fraction of the MeOH extract of the roots of *E. latifolius* [44].

Diab et al. recently reported GC–MS analysis of ethanol extract of the *E. spinosus* from Egypt, identifying 73 primary metabolites, including a monoterpene Z-3,7-dimethyl-2-octene [72].

### 3.3. Flavonoids

Flavonoids are low molecular weight, bioactive polyphenols that play a vital role in photosynthesis. Flavonoids are secondary metabolites and, as a class of natural compounds, have attracted attention due to their diverse pharmacological activities, particularly their antioxidant properties. Strong antioxidants, such as flavonoids, can shield the body from harmful free radicals. They accomplish this by scavenging free radicals, which is made possible by their capacity to donate hydrogen ions. Flavonoids also possess antifungal, antibacterial, and anti-inflammatory properties; additionally, they can protect the gastrointestinal mucosa from harm caused by necrotic agents and different ulcer models. Furthermore, flavonoids possess anti-carcinogenic properties because they can prevent cancer from developing and spreading by influencing angiogenesis, apoptosis, cellular differentiation, proliferation, and metastasis.

To the best of our knowledge, there is comparatively little research on the flavonol and flavone contents of Echinops species [73,74]. The genus Echinops is in a medium position in the evolutionary advancement of Asteraceae [75], with a flavone/flavonol ratio of 1/1.

The majority of flavonoids in the genus *Echinops* are flavones, extracted primarily from the entire plant and the members’ aerial portions. Apigenin, the most prevalent flavonoidal aglycone, has been isolated from the flower and entire *E. niveus* [64], *E. echinatus* [76], *E. integrifolius* [66], *E. spinosus* [77], and *E. albicaulis* [47] plant (Table 3).

*Echinops niveus* [64], *Echinops echinatus* [84], and *Echinops giganteus* [65] have all been reported to contain luteol (Table 3), while β-sitosterol-3-glucoside and stigmasterol have been isolated from *Echinops giganteus* [65], *Echinops ritro* [53], and *Echinops transiliensis* [41].

A new isoflavone glycoside, echinoside, along with 7-hydroxyisoflavone, kaempferol-4′-methylether, kaempferol-7-methylether, myrecetin-3-O-a-L-rhamnoside, kaempferol, and kaempferol-3-O-a-L-rhamnoside (Table 3), has been isolated from the entire *Echinops echinatus* plant [74].

Senejoux et al. reported, for the first time, 6-methoxyflavones in *Echinops integrifolius*, including jaceidin, centaureidin, hispidulin, and axillarin [66].

Trihydroxy methoxy flavone, kaempferol-3-*O*-methyl ether, and quercetin are the three main flavones in *Echinops taeckholmiana* Amin; they were first reported by Hamdan [61].

### 3.4. Alkaloids

Because of their distinct and varied pool of secondary metabolites, including phenolics, sesquiterpene lactones, alkaloids, and triterpenes, plants in the *Asteraceae* family have been shown to have important therapeutic uses. More than 14 species, including *E. echinatus*, *E. ritro*, and *E. sphaerocephalus*, have been shown to contain simple quinoline alkaloids in their aerial and/or subterranean portions [58,85,86].

**Figure 3:**
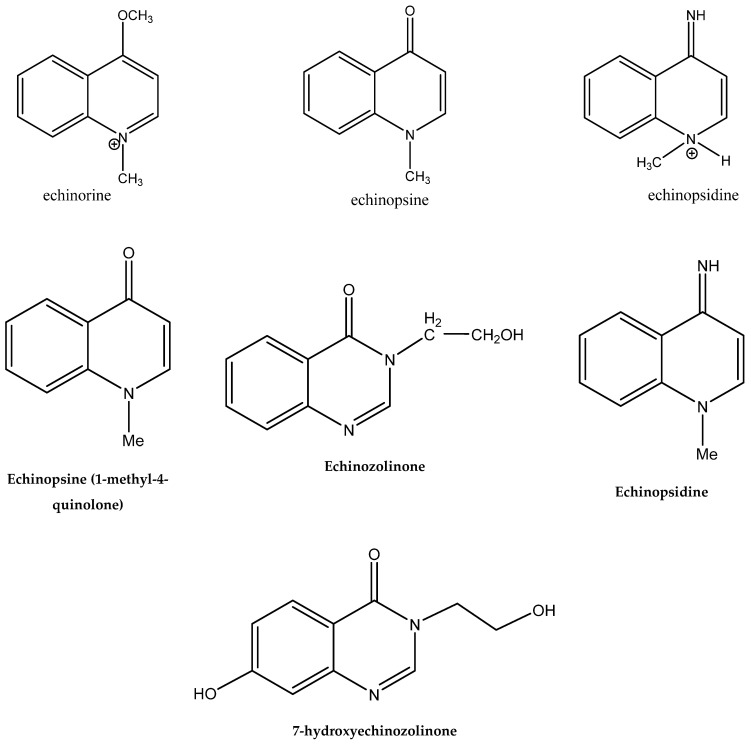
Alkaloids present in *Echinops*.

Alkaloids isolated from various *Echinops* spp. were found to be of the quinoline type, primarily 1-methyl-4-quinolone (Echinopsine) [87]. However, research on alkaloids is quite preliminary [88]. Echinopsine, a quinoline alkaloid, is used in traditional Chinese medicine to treat deep-seated breast carbuncles, ulcers, sodoku, and breast milk stoppage. The biological activity of echinopsine is still unclear, despite extensive research on the bioactivity of *Echinops sphaerocephalus* L. extracts [89]. The echinopsine moiety has the potential for broad-spectrum biological actions, as evidenced by the herbicidal, insecticidal, bactericidal, anti-tumor, antifungal, and antifeedant properties of a range of natural alkaloids containing it [90]. Echinopsine was found to possess activity against tobacco mosaic virus, a single-stranded RNA virus in the family *Togaviridae* [91]. Chaudhuri found that the aerial sections of *E. echinatus* contain echinopsine, as well as echinozolinone and echinopsidine, the first alkaloids to be isolated from *Echinops* spp. [92]. The same plant’s blooms were later used to isolate 7-hydroxyechinozolinone, another alkaloid [85](Figure 3). The alkaloids were in their glycosidic forms. The plants’ aerial portions were the primary source of the alkaloids. The most common alkaloid isolated from four distinct species—*E. echinatus*, *E. nanus*, *E. albicaulis*, and *E. orientalis*—was echinopsine [80,87,88,92].

A thorough analysis of the volatile components in the roots of *E. bannaticus* and *E. sphaerocephalus* led to the identification of 106 and 81 components, respectively, as part of our ongoing efforts to find potentially biologically active chemicals in medicinal plants. Two relatively uncommon chemical families were found in large concentrations in the oils: triquinane sesquiterpenoids (12.7 and 20.9%, respectively) and S-containing polyacetylene compounds (65.5 and 64.1%, respectively). *E. bannaticus* and *E. sphaerocephalus* have a strong relationship, and due to their high thiophene polyacetylene chemical concentration, *E. grijsii* may be included in the *Echinops* division alongside them. Strong relationships between essential oil constituents were identified using PCA, especially those found in the thiophene polyacetylene and triquinane sesquiterpenoid groups, which are very compatible with the biochemical routes thought to be responsible for the formation of these molecules [58].

According to Horn et al. and Patel et al. (2011), the oil properties of *E. sphaerocephalus* are comparable to those of other unsaturated plant oils, such as sunflower seed oil, soybean oil, or wheat germ oil [18,93,94]. The ratio of saturated to unsaturated acids and the content of fatty acids are useful markers for assessing the oil’s nutritional and functional worth. Because they are less resistant to rancidity and are more solid at room temperature, high unsaturated fatty acid oils are less common in industrial production, even though they are generally thought to be healthier than saturated fats and help lower blood cholesterol levels.

### 3.5. Therapeutic Potential of the Genus Echinops

**Figure 4:**
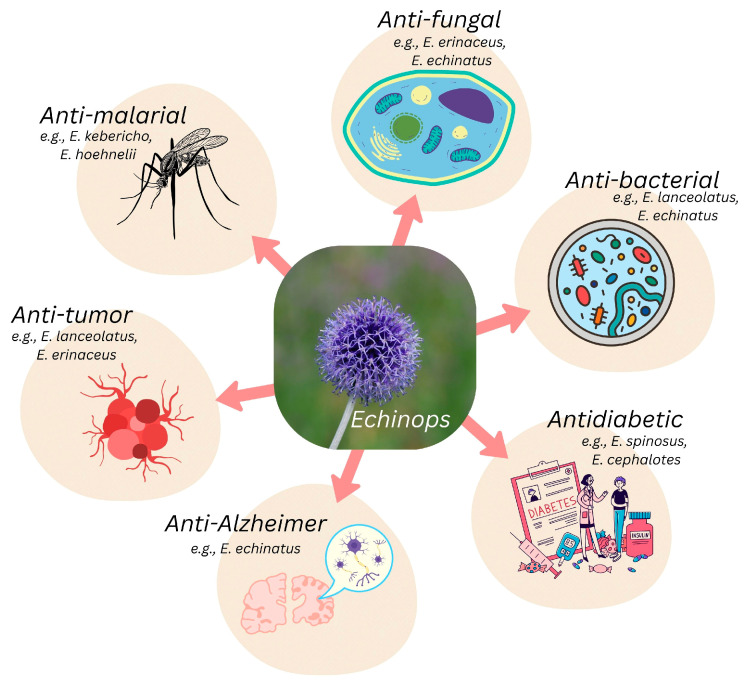
Summary of reported biological activities of various *Echinops* spp., based on the available literature. Created with Canva (www.canva.com).

The chemical components and extracts isolated from the many species in this genus have a broad range of biological effects, as summarized in Figure 4.

The *Echinops* genus has advantages in both the medical and ecological fields. Potential anti-inflammatory, antibacterial, and analgesic properties of the bioactive chemicals derived from *Echinops* extracts have recently been determined. These results suggest the genus’s significance in plant-based drug development and ethnopharmacology [4].

Based on its chemical composition, *Echinops* species have long been utilized to cure a variety of illnesses, such as fever, heart and respiratory conditions, bacterial and fungal infections, and more [20]. In China, for example, the roots of *Echinops grijisii* Hance have been used to clear heat rash, expel miasma, and stimulate milk secretion [30].

In vitro screening and analysis of the phytochemical properties of *Echinops* spp. have identified antimicrobial, antioxidant, and immunomodulatory therapeutic properties [4].

Antifungal, antibacterial, cytotoxic, antimalarial, and insecticidal pharmacological activities are defined by the presence of thiophenes, while anti-inflammatory, antioxidant, and hepato-protective properties are attributed to terpenes, flavonoids, and other phenolic substances [20,57].

#### 3.5.1. Antioxidant Activity

One of the main pathophysiological mechanisms of many liver illnesses is oxidative stress. Antioxidants from different natural sources have offered clinical promise in the treatment of liver diseases. Oxidative stress can result from liver injury as well as be the cause of hepatocyte damage, malfunction, and cell death. This dichotomy necessitates elucidating the causal relationship through experimental investigations of the hepatotoxic mechanisms of pro-oxidants, such as medications, and particular preventative and therapeutic strategies involving the administration of antioxidants derived from plants. Both the potential hepatoprotective ability of plant extracts in humans and the potential mechanisms of injury should be assessed in experimental models that use several biomarkers to evaluate hepatotoxicity. This strategy improves the likelihood that liver protection seen in animal and cell culture models may be effectively extended to human pathophysiology and treatment.

It has been suggested that the generation of ROS is a precursor to drug-induced hepatotoxicity and a sign of a drug’s potential for hepatotoxicity [85]. Numerous medications have been found to cause oxidative stress, which includes a rise in lipid peroxidation and cellular oxidants, a reduction in the liver’s antioxidant reserves, and a drop in the activity of antioxidant enzymes [37].

In addition to liver disease, oxidative stress is a key pathophysiological process in many other chronic diseases that are relevant to society. In experimental pharmacology, a variety of physiologically active chemicals and secondary metabolites derived from plants, primarily phenolic compounds, are being studied as sources of antioxidants that are essential for preventing disorders linked to oxidative stress. By giving lipids or lipid peroxyl radicals hydrogen atoms, flavonoids have been shown to have the ability to break free radical chains in lipids [95].

The antioxidant activity assessments of *Echinops* spp. roots are currently insufficient.

Kiyekbayeva et al. evaluated the antioxidant activity of the aqueous methanolic extract of *E. albicaulis* aerial parts by determining the reactive oxygen species (ROS) levels in active cultures of mononuclear cells from the peripheral blood of healthy adults [47]. The authors found that methanolic extract at concentrations of 50, 10, and 1 mg/mL significantly reduced ROS generation in the active cell cultures (3208, 3242, and 3188 μM H_2_O_2_, respectively) compared to the reference compound N-acetylcysteine. Higher concentrations (100, 500, and 1000 mg/mL) of aqueous methanolic extract of *E. albicaulis* induced overproduction of ROS, which may indicate drug-induced oxidative stress as a mechanism of toxicity [96]. The authors found that at a concentration of 1 mg/mL, the extract significantly (*p* < 0.001) reduced intracellular ROS production induced by hydrogen peroxide in cell cultures to 6926 μM H_2_O_2_, which is more than the reference antioxidant N-acetylcysteine (7378 μM H_2_O_2_); at higher concentrations (10 and 50 mg/mL), it showed antioxidant activity (7203.67 and 7768.67 μM H_2_O_2_, respectively) comparable to the reference N-acetylcysteine.

Erenler et al. investigated the antioxidant potential of chemical compounds isolated from Echinops orientalis Trauv. [57]. The authors found that seed and leaf extracts have high DPPH and moderate ABTS radical scavenging activities due to the isolated flavones, especially apigenin and its 7-O-glucoside, which exhibit high cation radical scavenging activities.

Quinic acid, a major compound in *E. ritro* leaves, exerted pronounced dose-dependent antioxidant activity in a cell model of H_2_O_2_-induced oxidative stress, restoring MDA levels [97].

3,5- and 4,5-dicaffeoylquinic and chlorogenic acid also have very strong antioxidant effects [98]. Caffeoylquinic acids exhibited a radical scavenging activity similar to that of ascorbic acid. They chelated Fe^2+^ and Cu^2+^ and disrupted chain reactions. Chlorogenic acid can scavenge different radicals and protect DNA from damage caused by oxidative stress.

Saida Hanane Zitouni-Nourine et al. recently studied *Echinops spinosissimus* Turra from Algeria. The authors investigated the total phenolic content and antioxidant properties of the root methanolic extract. The total phenolic content was equal to 95.31 ± 2.90 mg GAE/g DW, while the number of flavonoids was 16.01 ± 0.16 mg CE/g DW. The methanolic extract was found to exhibit antioxidant activity towards the DPPH radical, with an IC_50_ of 7.99 ± 0.28 mg/mL and a TAC of 30.30 ± 0.54 mg AAE/g DW [54]. Comparatively, previously investigated methanolic extract of *E. giganteus* root showed an in vitro free radical scavenging effect of 12.54 mg equivalent weight of Trolox per 100 g [99].

Extracts from *E. ritro* were studied by Zheleva-Dimitrova et al. and were found to effectively restore liver function, reduce oxidative stress, and diminish liver damage.

The presence of flavonoids, phenylethanoid glycosides, and different hydroxybenzoic, hydroxycinnamic, and acylquinic acids in the flowering head and leaf extracts led the authors to conclude that the *E. ritro* extracts can be employed as antioxidants in medicinal applications and to promote health [53].

#### 3.5.2. Antidiabetic Properties

**Figure 5:**
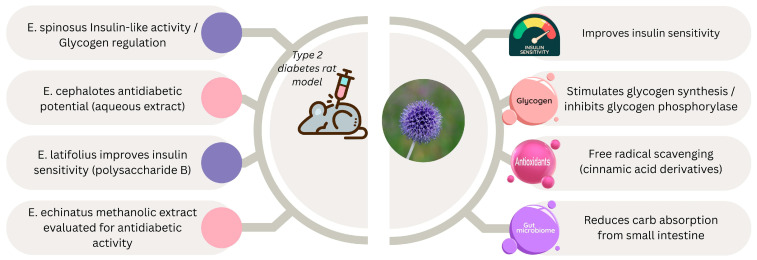
Antidiabetic mechanisms and phytochemical effects of selected *Echinops* species in experimental models of type 2 diabetes. Created with Canva (www.canva.com).

Numerous variables contribute to type 2 diabetes, a complex metabolic disease. The World Health Organization estimates that this illness affects over 422 million people and results in over 1.6 million fatalities per year. Eight out of every 1000 people are thought to have type 2 diabetes, and as people age, their risk of the disease rises. The number of children and adolescents receiving a diagnosis of this illness has increased in recent years [100]. Plants contain a variety of bioactive phytochemical substances that are thought to have positive health effects (Figure 5) [101,102]. The antidiabetic impact of the methanolic extract of several *E. echinatus* components has been assessed [88].

The 70% hydro-alcoholic root extract of *E. echinatus* was reported to have significant antidiabetic activity [103]. Alloxan-induced diabetic rats were investigated. After 21 days of therapy, the rats treated with the extract had lower blood glucose levels (164 mg/dL) compared with the negative control (277.6 mg/dL). Furthermore, the extract demonstrated the capacity to restore normal glomerular and distal convoluted tubule structure in the kidneys, as well as pancreatic islet cell regeneration; additionally, the extract was able to raise high-density lipoprotein levels, while lowering blood cholesterol, serum triglycerides, serum low-density lipoprotein, serum very low-density lipoprotein, and serum alkaline phosphate levels [104].

Aqueous methanolic extract of *Echinops echinatus* roots had effective hypoglycemic and antihyperglycemic agents in experimental models of type I and type II diabetes. The extract significantly improved glucose tolerance at all time points compared to fructose-fed rats at doses of 100, 300, and 500 mg/kg. The extract also corrected dyslipidemia associated with alloxan-induced diabetes [105].

Total *E. spinosus* extract and its flavonoid fraction showed promising antidiabetic activity [101]. The authors investigated the antidiabetic properties of both the total extract and its high flavonoid fraction in experimental diabetes induced by streptozotocin injection in rats. Seven days after streptozotocin administration, the diabetic animals were treated daily with total extract, high-flavonoid extract, or metformin as a standard antidiabetic drug for 28 days. The authors found that both total and high-flavonoid extracts demonstrated antidiabetic properties, as evidenced by lower glucose levels and increased levels of insulin, insulin receptor expression rate, and glycogen synthesis. Additionally, both extracts alleviated diabetic complications in the kidneys and liver by decreasing oxidative stress, modulating inflammatory mediators, suppressing the apoptotic cascade, and correcting diabetic dyslipidemia.

K. Benrahou et al. [106] evaluated the antidiabetic enzymatic activity of aqueous and ethanolic extracts of *E. Spinosus* roots using in vitro and ex vivo assays. The results showed that *α*-amylase, *α*-glucosidase, and lipase were effectively inhibited by the macerated ethanolic extract, with IC_50_ values of 371 ± 0.62, 18.6 ± 1.2, and 10.44 ± 1.08 μg/mL, respectively. The aqueous extract, on the other hand, was less potent against the three enzymes, with IC_50_ values of 668.8 ± 1.45, 19.68 ± 0.46, and 24.96 ± 1.52 μg/mL, respectively. However, both aqueous and ethanolic extracts significantly lowered blood sugar to 0.96 g/L and 0.93 g/L, respectively, after 90 min.

The terpenoidal components of *E. spinosus* demonstrate an insulin-like effect and promote intracellular glycogen deposition through the stimulation of glycogen production and inhibition of glycogen phosphorylase. It also improves glycogen metabolism when hepatic glycogen levels are low.

The aqueous extract of *E. cephalotes* has also been found to possess antidiabetic potential [107]. Another significant family of *Echinops* metabolites with antidiabetic properties is polysaccharide B, which has been isolated from *E. latifolius* Tausch. It was found to reduce levels of free fatty acids and triglycerides, improve insulin sensitivity, avoid hepatic metabolic abnormalities, and promote glucose intake and glycogen synthesis in IR-HepG2 cells [87].

Due to the presence of cinnamic acid and its derivatives, *Echinops* spp. can scavenge free radicals, boost the expression of glucose transporters, regulate or inhibit enzymes involved in glucose metabolism, and restore beta cell function [106].

#### 3.5.3. Anti-Osteoporosis Efficacy of *Echinops latifolius*

Mongolian medicine *Echinops* or *Echinops latifolius* Tausch was found to prevent postmenopausal osteoporosis. An osteoporosis model was established via ovariectomy in rats. Rats were treated with *Echinops* (16.26, 32.5, or 65 mg/kg/day) for 3 months. Results showed that *Echinops* significantly increased trabecular interconnectivity, thickness of trabeculae, and the connection of trabeculae. *Echinops* significantly increased bone mineral density and E2, but significantly reduced ALP and testosterone in dose-dependent manners. The authors concluded that Mongolian *Echinops* reduced bone loss, delayed the occurrence and development of osteoporosis, and increased ERα, ERβ, p-AKT, and P-ERK in bone marrow-derived stem cells [108].

**Figure 6:**
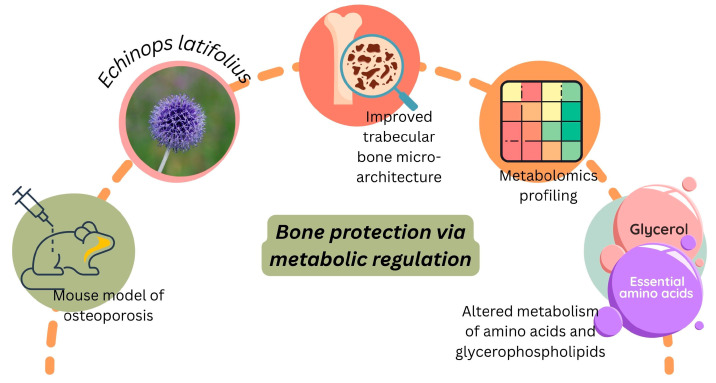
*Echinops latifolius* improved bone microstructure in ovariectomized rats by modulating amino acid and glycerophospholipid metabolism. Created with Canva (www.canva.com).

Later, Wang et al. [89] examined *Echinops latifolius* Tausch and its influence on the trabecular micro-architecture of ovariectomized rats by interfering with the metabolism of amino acids and glycerophospholipids, as determined by metabolomics profiling (Figure 6).

The authors observed a severe impairment of the bone micro-architecture of ovariectomized rats after administration of *Echinops latifolius*. In comparison with the control group, the morphologic parameters, including bone surface to bone volume and trabecular separation, increased significantly in ovariectomized rats (*p* < 0.01), while bone volume fraction, trabecular thickness, and trabecular number decreased significantly.

#### 3.5.4. Alzheimer’s Disease Prevention

Alzheimer’s disease is one of the most prevalent forms of age-related neurodegenerative dementia affecting elderly individuals worldwide. It is estimated that 13% of individuals over 65 and 45% of those over 85 suffer from Alzheimer’s syndrome [90,91].

**Figure 7:**
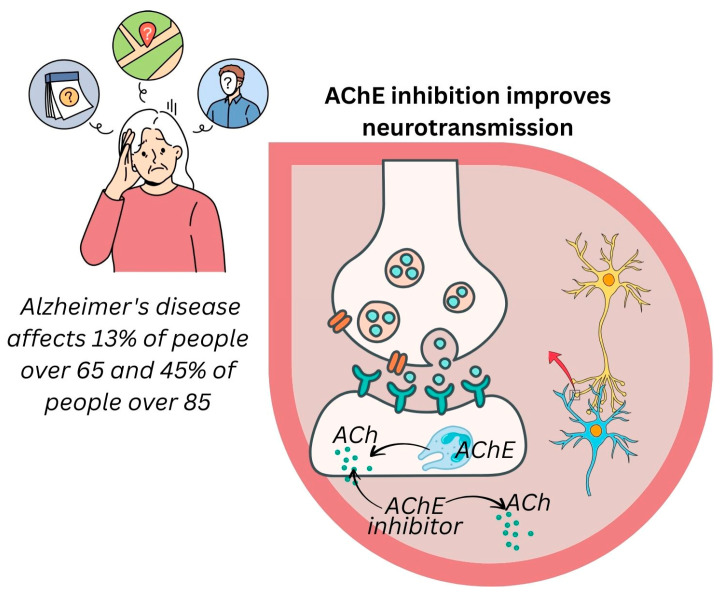
Anti-Alzheimer’s potential of *Echinops* species via cholinesterase inhibition. Created with Canva (www.canva.com).

Acetylcholinesterase (AChE) inhibition is one of the mechanisms through which anti-Alzheimer’s medications work. In one study, extracts from *E. ritro* were examined for their anti-cholinesterase (AChE and butyrylcholinesterase, BChE) activity (Figure 7).

*E. echinatus* and *E. ritro* were found to inhibit AChE, compared to galanthamine and physostigmine as standards [80,92]. Different extracts of leaves, stems, flowers, and achenes of *E. echinatus* were compared to galanthamine and physostigmine as standards. The methanol and ethyl acetate extracts showed the strongest AChE and BChE inhibitors. Ethyl acetate extracts of stems and leaves, on the other hand, strongly inhibited AChE, with IC_50_ values of 15.3 and 15.8 μg/mL, compared to physostigmine and galanthamine, which had IC_50_ values of 0.05 and 2.1 μM/mL, respectively. Moreover, the ethyl acetate extracts of the leaves and stems were found to possess the most potent inhibitory effect against BChE, with IC_50_ values of 17.5 and 16.3 μg/mL, compared to physostigmine and galanthamine (IC_50_ values of 0.08 and 19.3 μM/mL, respectively).

#### 3.5.5. Antimicrobial Activities

The leaves and stems of *E. echinatus* contain flavonoid compounds, which have been found to have considerable antimicrobial and antibacterial properties [61]. The antimicrobial test zone of inhibition was nearly identical to the positive control’s (0.1 mg/mL for gentamycin). This supports the effectiveness of the genus’s plant species in treating a number of infectious diseases.

The antibacterial properties of the essential oils and ethanolic extract of the tuber of *E. kebericho*, along with its fractions, were investigated. Its essential oils demonstrated efficacy against the most harmful *Staphylococcus aureus*, *Enterococcus faecalis*, and *Klebsiella pneumoniae* [88,109]. The essential oils of *E. kebericho* demonstrated efficacy against methicillin-resistant *Staphylococcus aureus*, with MIC values ranging from 78.125 to 625 μg/mL. The ethyl acetate fraction exhibited the strongest efficacy against methicillin-resistant *Staphylococcus aureus*, with an MIC value of 39.075 μg/mL. The extracts were potent against *Enterococcus faecalis* and *Klebsiella pneumoniae,* with MICs of 78.125 μg/mL and 1250 μg/mL, respectively.

The chloroform fraction of *E.erinaceus* Kit Tan extract exhibited the highest activity against *S. Aureus*, with an MIC value of 312.5 μg/mL, whereas *E. fecalis* exhibited the highest sensitivity to the hexane fraction, with an MIC value of 156.2 μg/mL.

The antimicrobial activity of the methanolic extract of the aerial parts of *E. lanceolatus* was also examined against eight bacterial strains. The extract showed weak to moderate antibacterial activity. The ethyl acetate fraction, on the other hand, showed the highest activity, followed by dichloromethane, *n*-hexane, and butanol fractions, with MIC values ranging from 256 to 1024 µg/mL [110].

*Echinops ritro* L. essential oils exhibited an antibacterial effect, and antibiofilm and bacterial membrane disruption have been proposed as mechanisms of action [111]. The antibacterial effect of extracts from the roots of *Echinops ritro* L. was due to the presence of thiophenes. Thiophenes had an antibacterial effect against *S. aureus*, with an MIC value of 8 µg/mL [112,113]. The antibacterial effects of thiophenes against *E. coli* have also been described, with MIC values of 64, 32, 64, 64 and 8 µg/mL [37]. Thiophenes isolated from the roots of *Echinops ritro* L. have been reported to have significant antifungal activity against various fungal isolates. The most active thiophenes were 5-(but-3-en-1-ynyl)-2,2-bithiophene (IC_50_ 4.2 µM) against *Colletotrichum gloeosporioides*, α-terthiophene (IC_50_ 1.9 µM), and 5-(3,4-dihydroxybut-1-ynyl)-2,2′-bithiophene (IC_50_ 1.1 µM) against *C. fragariae* [25,29,114].

The antimicrobial activity of the methanolic extract of the flowering aerial parts of *E. erinaceus* was evaluated against six microorganisms: *Bacillus subtilis*, *Pseudomonas aeruginosa*, *Escherichia coli*, *Candida albicans*, and *Aspergillus niger*. The authors found that the methanolic extract exhibited high antibacterial activity against all tested bacteria, especially against *B. subtilis* (27.5 ± 0.7 mm inhibition zone), and it also showed strong antifungal activity against *C. albicans* (26 ± 1.41 mm inhibition zone) compared to streptomycin [115]. The chloroform extract was active against *B. subtilis* (20.5 ± 1.41 mm), *P. aeruginosa* (17.5 ± 1.41 mm), and *E. coli* (18 ± 1.41 mm), but it had no activity against the fungal strains.

#### 3.5.6. Insecticidal Properties

In Ethiopia, burning the dry roots of *E. kebericho* produces smoke that serves as a natural insect repellent, keeping mosquitoes away [116].

The roots of *E. ellenbeckii* and *E. longisetus* exhibit very strong anthelmintic activity. Findings from an earlier study [117] also suggested that the traditional uses of these two and most likely other *Echinops* spp. in the treatment of intestinal worm infestation had a scientific foundation.

**Figure 8:**
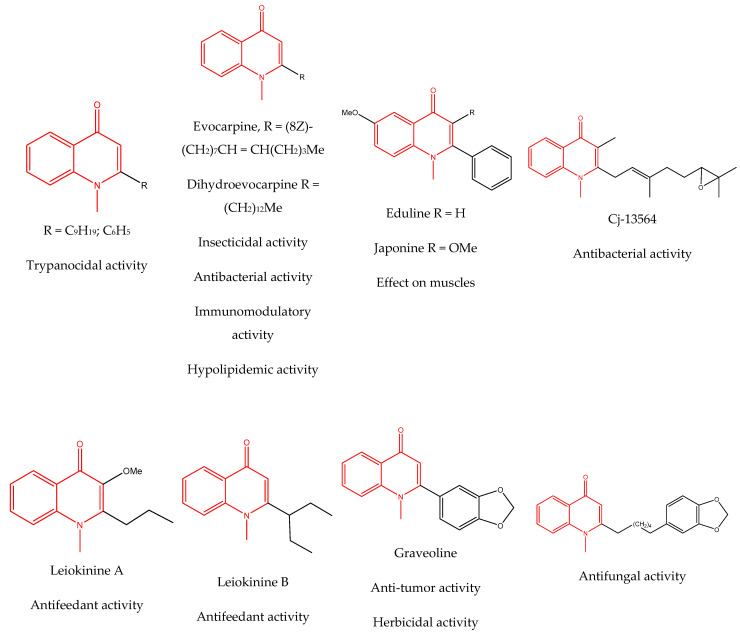

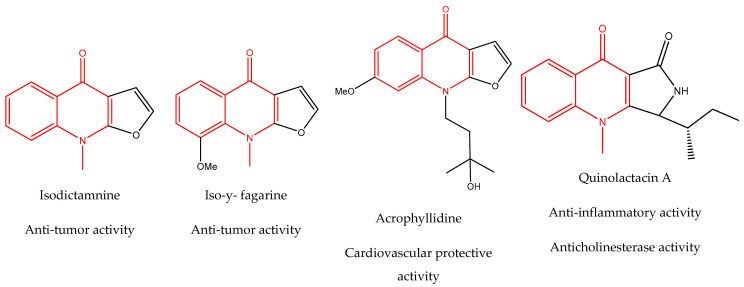
Biological activities of echinopsine derivatives.

A range of echinopsine derivatives with an acylhydrazone functional group that exhibit strong bactericidal, herbicidal, and insecticidal properties were synthesized by Cui et al. [118] (Figure 8).

The authors synthesized derivatives with benquinox [119], saijunmao [120], metaflumizone, and diflufenzopyr biological activities and echinopsine’s moiety. Cui et al. [118] found that the acylhydrazone derivatives have very good insecticidal potential against *Lepidoptera* pests, including fall armyworm (*Spodoptera frugiperda*), cotton bollworm (*Helicoverpa armigera*), corn borer (*Ostrinia nubilalis*), oriental armyworm (*Mythimna separata*), and diamondback moth (*Plutella xylostella*), compared to echinopsine. *Echinops* spp. also exhibited antimolluscicidal activity; thus, this plant is used to control snails that spread schistosomiasis in Ethiopia [121,122].

#### 3.5.7. Anti-Malarial Activity

**Figure 9:**
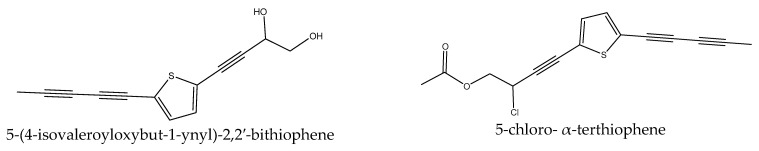
Thiophenes with antimalarial activity.

The methanolic extract of *E. kebericho* Mesfin’s rhizomes showed antiplasmodial efficacy against the rodent malaria parasite, *P. berghei*. This activity was attributed to the identified sesquiterpenes [123]. In a related investigation, various fractions of *E. kebericho* roots were shown to be active against Plasmodium berghei [124]. Bitew et al. concluded that the activity was due to the thiophenes in the *E. hoehnelii* root extract. Additional research is still needed to determine how well other species in the genus *Echinops* prevent malaria. Based on the currently available limited information, thiophene chemicals such as (18) and (19) are linked to the antimalarial activity of the genus *Echinops* (Figure 9); however, their mechanism of action remains to be elucidated.

#### 3.5.8. Cytotoxicity

**Figure 10:**
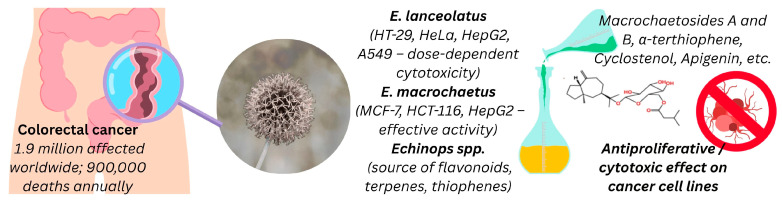
Anticancer potential of *Echinops* species and isolated bioactive compounds against human cancer cell lines. Created with Canva (www.canva.com).

Flavonoids (apigenin), terpenes (macrochaetosides (A and B), cyclostenol, erinaceosin), and thiophenes (α-terthiophene) are among the anti-tumor secondary metabolites found naturally in the genus *Echinops*. Several studies have demonstrated the anticancer activity of various *Echinops* species against cancer cell lines. The most common activity is against colonic carcinoma, one of the most common and severe diseases, especially in industrialized countries. It is estimated that colorectal cancer affects 1.9 million people worldwide and kills approximately 900,000 annually [125]. Colorectal cancer makes up 3.47% of cancer cases in men and 3% in women [126,127]. *Echinops* species exhibit notable anticancer activity, as illustrated in Figure 10.

Antiproliferative properties of the methanolic extract and fractions of aerial parts of *E. lanceolatus* were evaluated against HepG2 (human liver cancer cell line), HeLa (cervical cancer cells), HT-29 (human colon cancer cell line), and A549 (adenocarcinomic human alveolar basal epithelial cells). The results demonstrated that the ethyl acetate fraction suppressed cancer cell proliferation in a dose-dependent manner at doses ranging from 0.82 to 200 µg/mL. The extract exhibited strong cytotoxicity against A549 (IC50 8.27 µg/mL) and moderate cytotoxicity against HeLa (IC50 28.27 µg/mL) [110].

*E. latifolius* Tausch., especially four thiophens isolated from its extract, namely 5-(3,4-dihydroxybut-1-ynyl)-2,2′-bithiophene, 5-(4-hydroxy-1-butynyl)-2,2′-bithiophene, 5-{4-[4-(5-pent-1,3-diynylthiophene-2-yl)-but-3-yny}-2,2′-bithiophene, and 5-(4-hydroxybut-1-one)-2,2′-bithiophene displayed cytotoxic activity (IC_50_ values of 5.2, 10.2, 3.1, and 6.5 µmol/L) against human malignant melanoma (A375-S2) and human cervical carcinoma (HeLa) cell lines [42].

The dichloromethane fraction of *E*. *grijisi* showed activity against HepG2 (IC_50_ = 2 µg/mL) due to the presence of 5-(4-isovaleroyloxybut-1-ynyl)-2,2′-bithiophene and 5-(3-acetoxy-4-isovaleroyloxybut-1-ynyl)-2,2′-bithiophene, while 5-(prop-1-ynyl)-2-(3,4-diacetoxybut-1-ynyl)-thiophene showed activity against myeloid leukemia HL-60 (IC_50_ = 8 µg/mL) [43]. Zhang et al. evaluated the cytotoxic effect of thiophenes isolated from *E. grijisii* on human acute myeloid leukemia (HL60) and human chronic myelogenous leukemia (K562) cell lines. Significant effects were observed for 5-(4-hydroxybut-1-ynyl)-2-(pent-1,3-diynyl)-thiophene (IC_50_ 0.23 and 0.47 µg/mL, respectively) and 5-(penta-1,3-diynyl)-2-(3,4-dihydroxybut-1-ynyl)-thiophene (IC_50_ 0.27 and 0.43 µg/mL, respectively) [30].

The methanolic extract from the underground part of *E. giganteus* also exhibited cytotoxic activity against prostate cancer (Mia PaCa2) and two leukemia cells (CCRF-CEM and CEM/ADR5000) with IC_50_ values of 9.84, 6.68, and 7.96 µg/mL, respectively [128].

*E. macrochaetus* extracts were found to be active against human breast cancer cell line MCF-7 (IC_50_ = 2.1 and 0.18 μM), HepG2 (IC_50_ = 2.9 and 3.3 μM), and HCT-116 (IC_50_ = 3.6 and 2.3 μM) due to the presence of macrochaetoside A and cyclostenol [63].

#### 3.5.9. Food Supplement

The market must constantly expand and adapt to accommodate new food product sources and make the best use of existing resources due to the rising demand for food. *Echinops sphaerocephalus*, a plant that has long been used in beekeeping to provide bees with a consistent supply of nectar, may be one such resource. The oil content was recently examined to determine whether it would be appropriate for use as a culinary oil source. Its seeds, which are mainly composed of extremely important unsaturated linoleic fatty acids, have been found to contain up to 25% oil.

*E. sphaerocephalus* is considered a desirable culture for honey farms because of its high pollen and nectar content [19]. Its tube-shaped blooms readily draw pollinators to its aroma and yield 2–6 mg of honey apiece. In central and southern Europe, it is a common crop in honey production [94].

The term “mannas” refers to the sweet and sticky secretions that certain plants produce as a result of insect feeding, plant reactions to mechanical stimuli, or changes in the temperature outside of the plant tissues. The distribution of host and breeding insects, the period of production, and the manner of exploitation are some of the elements that contribute to the production of manna, even though the precise methods and conditions are still unknown. Several ecological and biological elements also contribute to the development and reproduction of manna-producing insects [13,129,130]. Mannas exhibit an array of general properties such as laxative, antipyretic, and cholagogue/choleretic effects [131,132,133]. Manna contains a large amount of cellulose (about 18%), and its syrup has been used in traditional medicine as a febrifuge and to treat severe coughs brought on by bronchial irritation [134]. Such findings highlight the multifaceted value of *Echinops sphaerocephalus* as a potential contributor to the food and pharmaceutical industries. Its diverse applications underscore the importance of further research aimed at optimizing its cultivation and sustainable utilization.

## 4. Conclusions

The genus *Echinops* represents a valuable source of structurally diverse secondary metabolites, particularly thiophenes, terpenes, flavonoids, and alkaloids, many of which are strongly correlated with its biological activities, such as antimalarial, anti-inflammatory, antidiabetic, cytotoxic, and neuroprotective effects. Current findings suggest that these compounds hold considerable promise for the development of novel therapeutic agents. However, the majority of studies remain at the in vitro or in vivo level, with limited clinical validation.

While preclinical studies support the use of Echinops species, further toxicological evaluations and clinical trials are essential to confirm efficacy and ensure the safe therapeutic applications of these species in modern medicine.

## Figures and Tables

**Table 1:** Structures of thiophenes present in *Echinops* L.

Name of the Secondary Metabolite	Chemical Structure	Isolated from	Reference
monothiophenes			
2-(Penta-1,3-diynyl)-5-(3,4-dihydroxybut-1-ynyl)thiophene	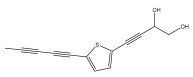	*E. grijsii* Hance	[22]
5-(penta-1,3-diynyl)-2-(3-chloro-4-hydoxy-but-1-ynyl)-thiophene	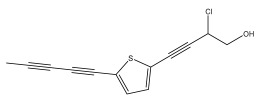	*E. ellenbeckii*	[23]
		*E. giganteus*	[20]
		*E. hispidus* Fresen.	[20]
		*E. longisetus*	[20]
		*E. macrochaetus*	[20]
2-(pent-3-en-1-ynyl)-5-(4-hydroxybut-1-ynyl)-thiophene	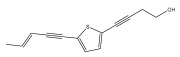	*E. pappii*	[24]
5-(4-hydroxybut-1-ynyl)-2-(pent-1,3-diynyl)-thiophene	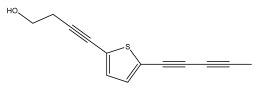	*E. pappii*	[24]
		*E. ritro*	[25,26,27]
		*E. grijsii*	[28,29,30]
		*E. giganteus*	[20,31,32]
5-(penta-1,3-diynyl)-2-(but-3-en-1-ynyl)-thiophene	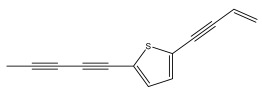	*E. ellenbeckii*	[33]
		*E. exaltatus* Schrad	[34]
		*E. humilis* Bieb.	[34]
		*E. niveus* Walb. ex Royle	[34]
		*E. orientalis* Trautv.	[34]
		*E. sphaerocephalus* L.	[34]
		*E. dahuricus* Fisch.	[34]
		*E. latijolius* Tausch.	[34]
		*E. leiopolyceras* Bomm.	[34]
		*E. microcephalus* Sibth. et Sm.	[34]
		*E. ruthenicus’*	[34]
		*E. spinosissimus* Turra	[34]
		*E. spinosus* L.	[34]
		*E. tschimganicus* B. Fedtsch.	[34]
		*E. exaltatus* Schrad	[34]
		*E. gmelini* Turcz	[34]
5-(penta-1,3-diynyl)-2-(4-acetoxy-but-1-ynyl)-thiophene	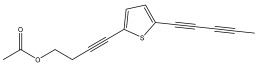	*E. ellenbeckii*	[33]
		*E. humilis* Bieb.	[34]
		*E. niveus* Walb. ex Royle	[34]
		*E. orientalis* Trautv.	[34]
		*E. sphaerocephalus* L.	[34]
		*E. dahuricus* Fisch.	[34]
		*E. latijolius* Tausch.	[34]
		*E. leiopolyceras* Bomm.	[34]
		*E. microcephalus* Sibth. et Sm.	[34]
		*E. ruthenicus’*	[34]
		*E. spinosissimus* Turra	[34]
		*E. spinosus* L.	[34]
		*E. tschimganicus* B. Fedtsch.	[34]
		*E. humilis* Bieb.	[34]
		*E. commutatus* Juratzka	[34]
		*E. gmelini* Turcz.	[34]
		*E. exaltatus* Schrad	[34]
5-(5,6-dihydroxy-hexa-1,3-diynyl)-2-(prop-1-ynyl)-thiophene (echinoynethiophene A)	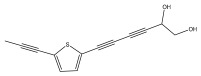	*E. grijsii*	[35,36]
		*E. grijsii*	[30]
5-(penta-1,3-diynyl)-2-(3,4-dihydroxybut-1-ynyl)-thiophene	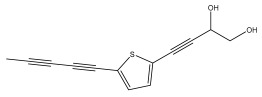	*E. grijsii*	[28,35,36]
		*E. ritro*	[37]
		*E. grijsii*	[22,29]
		*E. grijsii*	[30]
		*E. giganteus*	[31]
		*E. transiliensis*	[38]
		*E. hoehnelii*	[39]
Echinopsacetylenes B	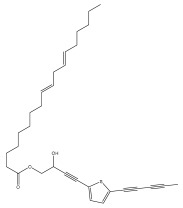	*E. transiliensis*	[40]
Echinothiophenegenol ((R)-5-hydroxy-6-((1E,3E)-6-hydroxyhexa-1,3-dien-1-yl)-2-(hydroxymethyl)thieno[2,3-e]isobenzofuran-8(6H)-one)	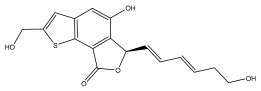	*E. grijsii*	[30]
		*E. nanus*	[41]
5-(4-acetoxy-3-chlorobut-1-ynyl)-2-(pent-1,3-diynyl)-thiophene	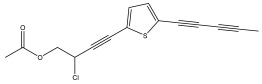	*E. ritro*	[25]
		*E. exaltatus* Schrad	[34]
		*E. humilis* Bieb.	[34]
		*E. niveus* Walb. ex Royle	[34]
		*E. orientalis* Trautv.	[34]
		*E. sphaerocephalus* L.	[34]
		*E. tschimganicus* B. Fedtsch.	[34]
		*E. exaltatus* Schrad	[34]
		*E. gmelini* Turcz	[34]
5-(prop-1-ynyl)- 2-(3,4-diacetoxybut-1-ynyl)-thiophene	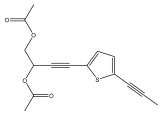	*E. latifolius*	[42]
		*E. grijsii*	[43]
5-(1,2-dihydroxy-ethyl)-2-(Z)-hept-5-ene-1,3-diynylthiophene	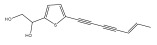	*E. latifolius*	[44]
5-(1,2-dihydroxyethyl)-2-(*E*)-hept-5-ene-1,3-diynylthiophene	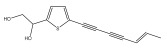	*E. latifolius*	[44]
5-(penta-1,3-diynyl)-2-(3-methoxy-4-hydroxy-but-1-ynyl)-thiophene	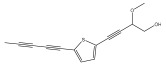	*E. hoehnelii*	[39]
5-(penta-1,3-diynyl)-2-(3-methoxy-4-acetoxy-but-1-ynyl)-thiophene	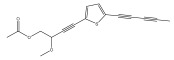	*E. hoehnelii*	[39]
2-(penta-1, 3-diynyl)-5-(3, 4-dihydroxybut-1-ynyl) thiophene	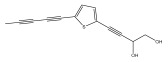		[22,45]
5-(penta-1,3-diynyl)-2-(3-acetoxy-4-hydroxy-but-1-ynyl)-thiophene	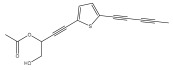	*E. transiliensis*	[38]
Junipic acid (5-(prop-1-yn-1-yl)thiophene-2-carboxylic acid)	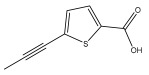	*E. ritro*	[37]
dithiophenes			
5-(but-3-en-1-ynyl)-2,2′-bithiophene (**1**)	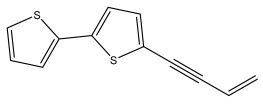	*E. macrochaetus*	[23]
		*E. exaltatus* Schrad	[34]
		*E. humilis* Bieb.	[34]
		*E. niveus* Walb. ex Royle	[34]
		*E. orientalis* Trautv.	[34]
		*E. sphaerocephalus* L.	[34]
		*E. dahuricus* Fisch.	[34]
		*E. latijolius* Tausch.	[34,46]
		*E. leiopolyceras* Bomm.	[34]
		*E. microcephalus* Sibth. et Sm.	[34]
		*E. ruthenicus’*	[34]
		*E. spinosissimus* Turra	[26,34]
		*E. spinosus* L.	[34]
		*E. tschimganicus* B. Fedtsch.	[34]
		*E. exaltatus* Schrad	[34]
		*E. gmelini* Turcz	[34]
		*E. pappii* Chiov.	[23]
		*E. ritro*	[25,27]
		*E. grijsii*	[28]
		*E. nanus* Bunge	[41]
		*E. albicaulis*	[26,47]
5-[(5-acetoxymethyl-2-thienyl)-2-(but-3-en-1-ynyl)]-thiophene	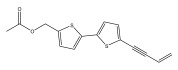	*E. ellenbeckii*	[33]
5-(3,4-dihydroxybut-1-ynyl)-2,2′-bithiophene	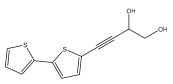	*E. grijsii*	[28,29,30,35,36]
		*E. ritro*	[37]
		*E. latifolius*	[42]
		*E. transiliensis*	[38]
2,2′-bithiophene-5-carboxylic acid	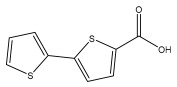	*E. grijsii*	[28,35]
		*E. ritro*	[37]
5-(3-buten-1-ynyl)-2,2′-bithiophene	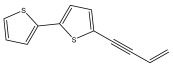	*E. grijsii*	[35]
5-(4-isovaleroyloxybut-1-ynyl)-2,2′-bithiophene	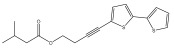	*E. grijsii*	[28,35]
		*E. grijsii*	[46]
		*E. grijsii*	[43]
		*E. grijsii*	[48]
		*E. ritro*	[25,26]
Grijisone A	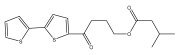	*E. grijsii*	[49]
5-(4-hydroxy-3-methoxy-1-butyny)-2,2′-bithiophene (4-([2,2′-bithiophen]-5-yl)-2-methoxybut-3-yn-1-ol)	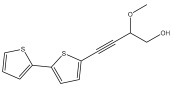	*E. grijsii*	[28]
1-([2,2′-bithiophen]-5-yl)ethan-1-one	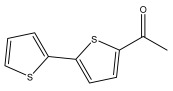	*E. latifolius*	[42]
		*E. grijsii*	[28]
		*E. ritro*	[37]
5-formyl-2,2′-bithiophene	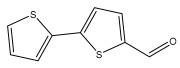	*E. grijsii*	[28]
5′-(3,4-Dihydroxybut-1-yn-1-yl)-[2,2′-bithiophene]-5-carbaldehyde	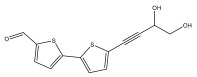	*E. ritro*	[37]
4-Hydroxy-1-(5′-methyl-[2,2′-bithiophen]-5-yl)butan-1-one	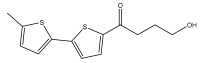	*E. ritro*	[37]
5′-(3,4-Dihydroxybut-1-yn-1-yl)-[2,2′-bithiophene]-5-carboxylic acid	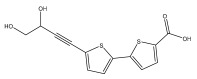	*E. ritro*	[37]
Methyl 2,2′-bithiophene-5-carboxylate	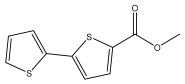	*E. grijsii*	[28]
5-(3-hydroxymethyl-3-isovaleroyloxyprop-1-ynyl)-2,2′-bithiophene	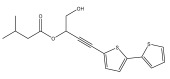	*E. latifolius*	[46]
		*E. grijsii*	[28]
5-(4-hydroxy-1-butynyl)-2,2′-bithiophene	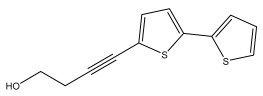	*E. latifolius*	[42]
		*E. ritro*	[25]
		*E. latifolius*	[42]
		*E. grijsii*	[28,30]
		*E. ritro*	[37]
		*E. ritro*	[26]
5-(4-acetoxy-1-butynl)-2,2′-bithiophene (4-([2,2′-bithiophen]-5-yl)but-3-yn-1-yl acetate)	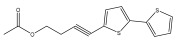	*E. grijsii*	[28]
5-(3-hydroxy-4-isovaleroyloxybut-1-ynyl)-2,2′-bithiophene	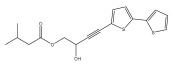	*E. latifolius*	[46]
5-(3-acetoxy-4-isovaleroyloxybut-1-ynyl)-2,2′-bithiophene	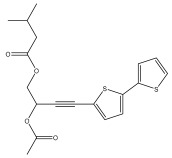	*E. latifolius*	[46]
5-(3,4-diacetoxybut-1-ynyl)-2,2′-bithiophene	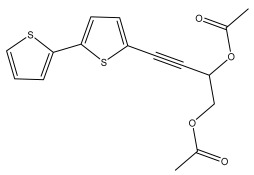	*E. ritro*	[25,26]
		*E. latifolius*	[34]
		*E. niveus* Walb. ex Royle	[34]
		*E. orientalis* Trautv.	[34]
		*E. gmelini Turcz.*	[34]
		*E. giganteum*	[34]
		*E. sphaerocephalus* L.	[34]
		*E. dahuricus* Fisch.	[34]
		*E. ruthenicus*	[34]
		*E. commutatus* Juratzka	[34]
		*E. spinosus* L.	[34]
		*E. tschimganicus* B. Fedtsch.	[34]
		*E. exaltatus* Schrad	[34]
		*E. gmelini* Turcz	[34]
		*E. grijsii*	[43]
		*E. grijsii*	[29]
		*E. grijsii*	[43]
		*E. transiliensis*	[38]
5-(4-hydroxybut-1-one)-2,2′-bithiophene (1-([2,2′-bithiophen]-5-yl)-4-hydroxybutan-1-one)	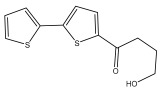	*E. latifolius*	[42]
		*E. ritro*	[37]
Methoxy-arctinol-b (2-methoxy-2-(3′-methoxy-5′-(prop-1-yn-1-yl)-[2,2′-bithiophen]-5-yl)ethan-1-ol)	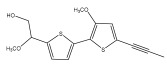	*E. latifolius*	[44]
Arctinol-b (1-(5′-(prop-1-yn-1-yl)-[2,2′-bithiophen]-5-yl)ethane-1,2-diol)	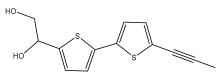	*E. grijsii*	[30,37]
		*E. latifolius*	[44]
		*E. ritro*	[37]
Arctinol a ((5′-(prop-1-yn-1-yl)-[2,2′-bithiophen]-5-yl)methanol)	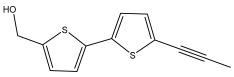	*E. latifolius*	[37,44]
		*E. ritro*	[37]
		*E. ritro*	[37]
Methyl [5′-(1-propynyf)-2,2′-bithienyl-5-yl] carboxylate	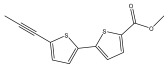	*E. latifolius*	[44]
5-(3-hydroxy-4-acetoxybut-1-ynyl)-2,2′-bithiophene (4-([2,2′-bithiophen]-5-yl)-2-hydroxybut-3-yn-1-yl acetate)	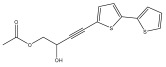	*E. transiliensis*	[38]
		*E. transiliensis*	[38]
5′-(3,4-dihydroxybut-1-yn-1-yl)-[2,2′-bithiophene]-5-carbaldehyde	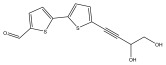	*E. ritro*	[37]
5′-(3,4-dihydroxybut-1-yn-1-yl)-[2,2′-bithiophene]-5-carboxylic acid	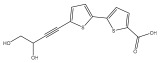	*E. ritro*	[37]
4-hydroxy-1-(5′-methyl-[2,2′-bithiophen]-5-yl)butan-1-one	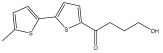	*E. ritro*	[37]
Arctinal (5′-(prop-1-yn-1-yl)-[2,2′-bithiophene]-5-carbaldehyde)	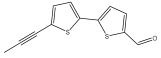	*E. ritro*	[37]
4-(5′-methyl-[2,2′-bithiophen]-5-yl)but-3-yn-1-ol	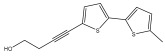	*E. ritro*	[37]
Arctic acid	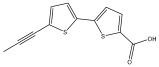	*E. ritro*	[37]
2,2-Dimethyl-4-[5-(prop-1-ynyl)-2,2-bithiophen-5-yl]-1,3-dioxolane	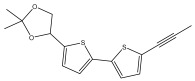	*E. spinosus*	[50]
terthiophenes			
α-terthiophene (**2**)	** 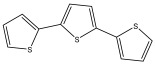 **	*E. ellenbeckii*	[23]
		*E. pappii*	[23]
		*E. niveus* Walb. ex Royle	[34]
		*E. orientalis* Trautv.	[34]
		*E. gmelini* Turcz.	[34]
		*E. giganteum*	[34]
		*E. sphaerocephalus* L.	[34]
		*E. dahuricus* Fisch.	[34]
		*E. ruthenicus*	[34]
		*E. commutatus* Juratzka	[34]
		*E. spinosus* L.	[34]
		*E. tschimganicus* B. Fedtsch.	[34]
		*E. exaltatus* Schrad	[34]
		*E. gmelini* Turcz	[34]
		*E. macrochaetus*	[23]
		*E. grijsii*	[28,48,51,52]
		*E. latifolius*	[34,46]
		*E. ritro*	[25,53]
		*E. ritro*	[26]
		*E. nanus*	[41]
		*E. albicaulis*	[25,26,47]
		*E. transiliensis*	[41]
		*Echinops spinosissimus* Turra	[34,54]
5-chloro- α-terthiophene	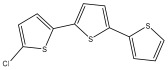	*E. grijsii*	[35,52]
5-acetyl α-terthiophene	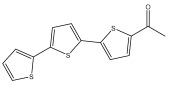	*E. grijsii*	[35,52]
5,5′-dichloro-α-terthiophene	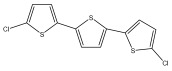	*E. grijsii*	[35,52]
Grijisyne A	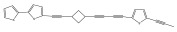	*E. grijsii*	[49]
5-{4-[4-(5-pent-1,3-diynylthiophen-2-yl)-but-3-ynyloxy]-but-ynyl}-2,2′-bithiophene	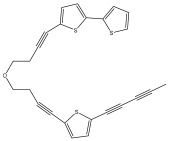	*E. latifolius*	[42]
Cardopatine	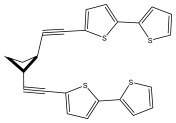	*E. grijsii*	[28,35,45]
		*E. latifolius*	[45,46]
		*E. ritro*	[25,26]
Isocardopatine	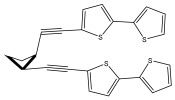	*E. grijsii*	[28,29,35,43]
		*E. ritro*	[25]
Echinopsacetylenes A	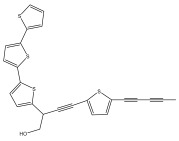	*E. transiliensis*	[40]

**Table 2:** Structures of the main terpenes found in *Echinops* spp.

Name of the Terpene	Chemical Structure	Isolated from	Reference
Dehydrocostus lactone	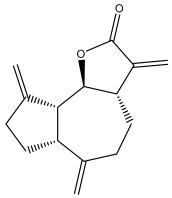	*E. amplexicauli*	[23]
		*E. kebericho*	[23]
Costunolide	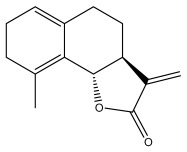	*E. amplexicaulis*,	[23,24]
		*E. kebericho*,	[23,24]
		*E. pappii*	[23,24]
Dihydrocostunolide	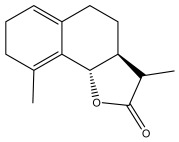	*E. amplexicaulis*	[23]
Echinopines A	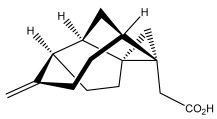	*E. spinosus*	[59]
Echinopines B	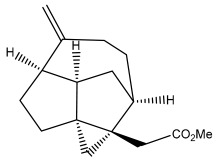	*E. spinosus*	[59]
(3α,4α,6α)-3,13-dihydroxyguaia-7(11),10(14)-dieno-12,6-lactone)	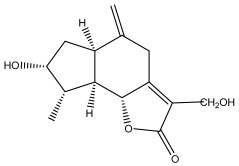	*E. ritro*	[60]
β-vatirenene	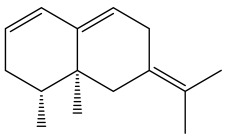	*E. taeckholmiana*	[61]
jatamol A	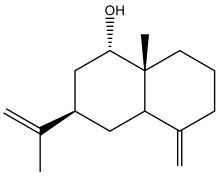	*E. taeckholmiana*	[61]
(3α,4α,6α,11ß)-3-hydroxyguai-1(10)-eno-12,6-lactone)	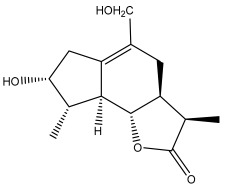	*E. ritro*	[60]
(11α)-11,13-dihydroarglanilic acid methyl ester	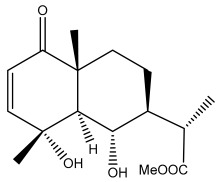	*E. ritro*	[60]
Vulgarin	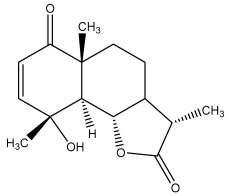	*E. ritro*	[60]
(3*R*,3a*S*,6a*R*,9*S*,9a*R*,9b*S*)-octahydro-3,9-dimethyl-6-methyleneazuleno[4,5-b]furan2,8(3*H*,9b*H*)-d ione	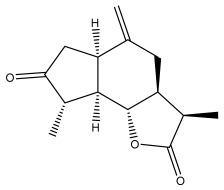	*E. ritro*	[60]
(3a*S*,6a*R*,8*S*,9*S*,9a*R*,9b*R*)-decahydro-8-hydroxy-9-methyl-3,6 dimethyleneazuleno[4,5-b]furan-2(9b*H*)-one	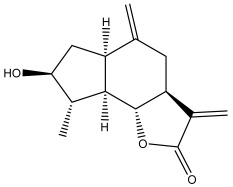	*E. ritro*	[60]
(3a*S*,6a*R*,8*R*,9*R*,9a*R*,9b*R*)-decahydro-8-hydroxy-3,3,9-trimethyl-6-methyleneazuleno[4,5-b]furan-2(9b*H*)-one	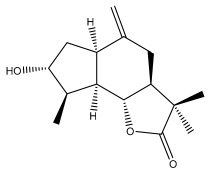	*E. ritro*	[60]
(3*R*,3a*S*,6a*R*,8*S*,9*S*,9a*R*,9b*S*)-decahydro-8-hydroxy-3,9-dimethyl-6-methyleneazuleno[4,5-b]furan-2(9b*H*)-one	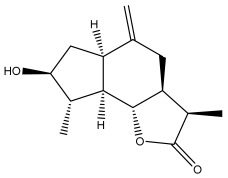	*E. ritro*	[60]
Santamarin	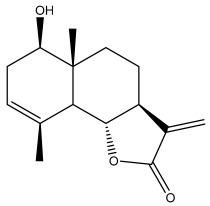	*E. pappii*	[24]
		*E. ritro*	[60]
Reynosin	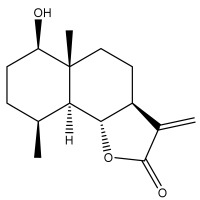	*E. pappii*	[24]
Caryophyllene epoxide	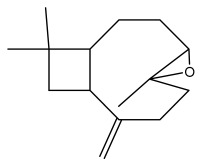	*E. giganteus*	[23]
		*E. hispidus*	[23]
Echusoside	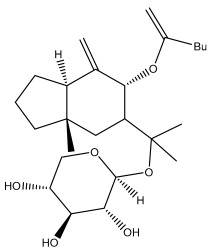	*E. hussoni* Boiss.	[62]
(3*S*,3a*S*,5a*R*,6*R*,8*R*,9b*S*)-decahydro-6,8-dihydroxy-3,5a-dimethyl-9-methylenenaphtho[1,2-b]furan-2(*9bH*)-one	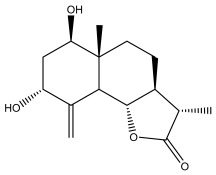	*E. ritro*	[60]
(3*S*,3a*S*,5a*R*,6*S*,9b*S*)-3,3a,4,5,5a,6-hexahydro-6-hydroxy-3,5a,9-trimethylnaphtho[1,2-b]furan-2,7(9a*H*,9b*H*)-dione	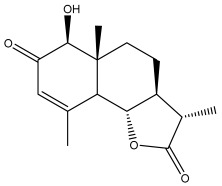	*E. ritro*	[60]
2,6,10-trimethyldodeca-2,6,10-triene	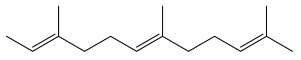	*E. albicaulis*	[47]
Macrochaetosides A	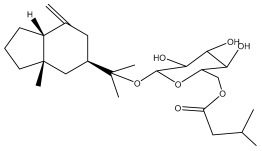	*E. macrochaetus*	[63]
Macrochaetosides B	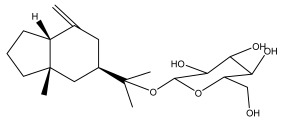	*E. macrochaetus*	[63]
Latifolanone A	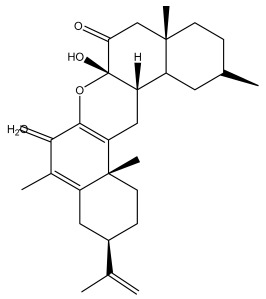	*E. latifolius*	[44]
Atractylenolide-II	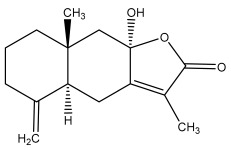	*E. latifolius*	[44]
ß-amyrin	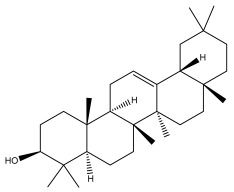	*E. niveus*	[64]
Betulinic acid	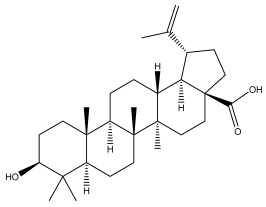	*E. niveus*	[64]
Lupeol	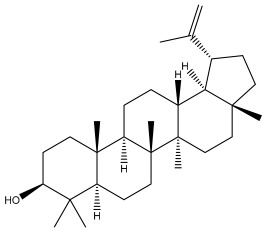	*E. niveus*	[64]
		*E. giganteus*	[65]
		*E. integrifolius*	[66]
		*E. echinatus*	[67]
Taraxasterol	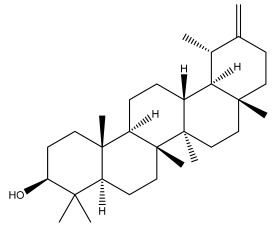	*E. niveus*	[64]
Taraxasterol acetate	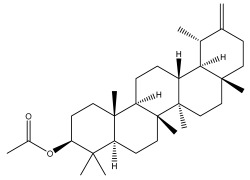	*E. niveus*	[64]
		*E. echinatus*	[68]
20-oxo-30-nortaraxast-21-en-3β-ol	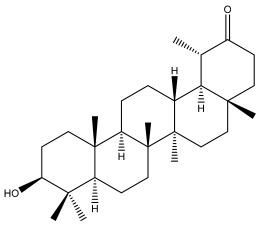	*Echinops latifolius* Tausch	[69]
Taraxeryl acetate	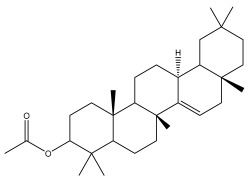	*E. taeckholmiana*	[61]
ß-sitosterol	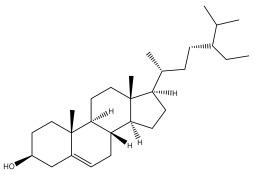	*E. niveus*	[64]
		*E. transiliensis*	[41]
		*E. giganteus*	[32]
		*E. orientalis*	[57]
ß-sitosterol glucoside	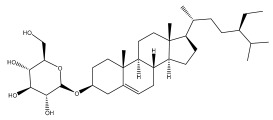	*E. niveus*	[64]
		*E. giganteus*	[65]
		*E. integrifolius*	[66]
		*E. albicaulis*	[47]
Reynosin	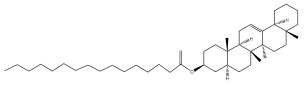	*E. pappii*	[24]
Gmeliniin A	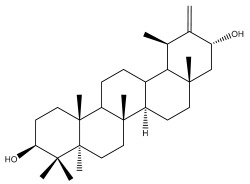	*E. gmelinii*	[70]
Stigmasterol	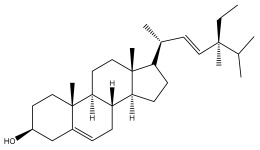	*E. transiliensis*	[41]
		*E. macrochaetus*	[63]
		*E. integrifolius*	[66]
		*E. giganteus*	[32]
Lupeol acetate	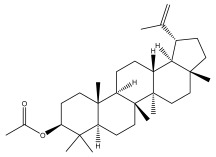	*E. integrifolius*	[66]
		*E. echinatus*	[67]
		*E. albicaulis*	[47]
Lupeol linoleate	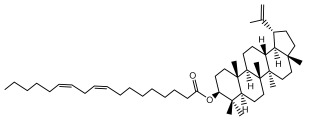	*E. albicaulis*	[47]
Ajugasterone C	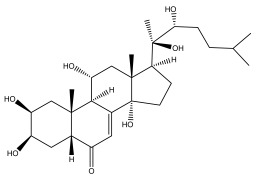	*E. grijisii*	[36]
Ursolic acid	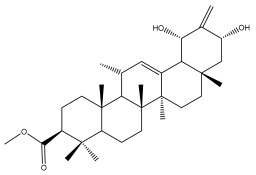	*E. giganteus*	[32]
Echinopsolide A (3ß-acetoxy-15α-bromoolean-13ß,28-olide)	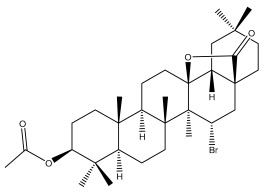	*E. giganteus*	[31]
ß-amyrin acetate	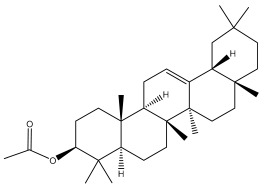	*E. giganteus*	[31]
3ß-acetoxy-taraxast-12,20(30)-diene-11α-21α-diol	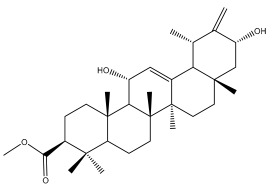	*E. galalensis*	[71]
α-amyrin	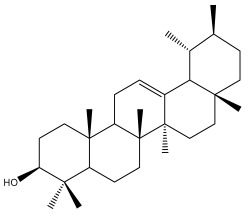	*E. galalensis*	[71]
Erythrodiol	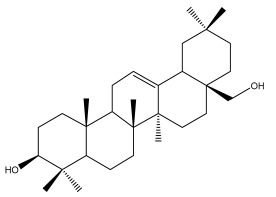	*E. galalensis*	[71]
Lup-20(29)-ene-1,3-diol	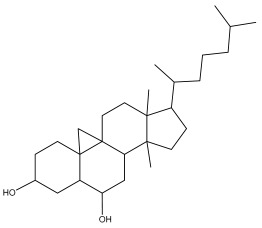	*E. galalensis*	[63]
Cyclostenol	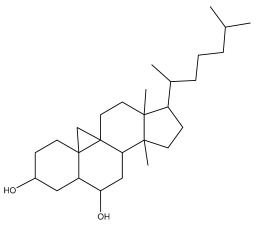	*E. macrochaetus*	[63]

**Table 3:** Flavonoids in *Echinops* spp.

Flavonoids and Other Phenolic Compounds	Chemical Structure	Isolated from	Reference
Apigenin	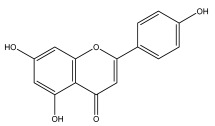	*E. niveus*	[64]
		*E. echinatus*	[76]
		*E. integrifolius*	[66]
		*E. spinosus*	[77]
		*E. albicaulis*	[47]
Luteolin	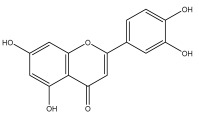	*E. niveus*	[64]
		*E. grijisii*	[36]
Nivegin (5,7-dihydroxy-4-(4-hydroxyphenyl)-2H-chromen-2-one)	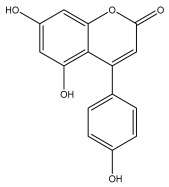	*E. niveus*	[64,78]
Nivetin (5,7-dihydroxy-4-(4-methoxyphenyl)-2H-chromen-2-one)	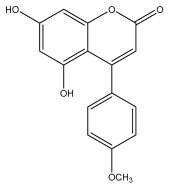	*E. niveus*	[73,79]
Apigenin 7-*O*-glucoside	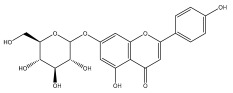	*E. echinatus*	[76]
		*E. spinosus*	[77]
		*E. orientalis*	[57]
		*E. ritro*	[80]
Echitin	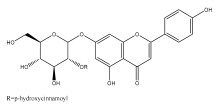	*E. echinatus*	[76]
Echinoside	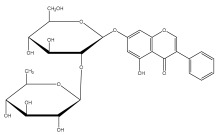	*E. echinatus*	[74]
7-hydroxyisoflavone	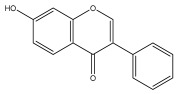	*E. echinatus*	[74]
Kaempferol	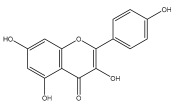	*E. echinatus*	[74]
Kaempferol-4′-methylether	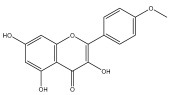	*E. echinatus*	[74]
Kaempferol-7-methylether	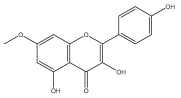	*E. echinatus*	[74]
Kaempferol-3-*O*-α-L-rhamnoside	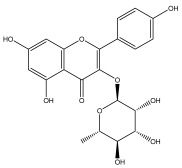	*E. echinatus*	[74]
		*E. heterophyllus*	[81]
Myrecetin-3-*O*-α-L-rhamnoside	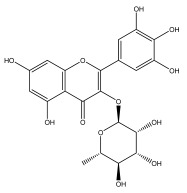	*E. echinatus*	[74]
Chrysoeriol	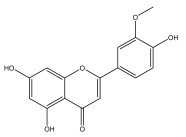	*E. integrifolius*	[66]
Hispidulin	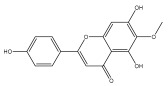	*E. integrifolius*	[66]
Jaceidin	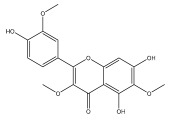	*E. integrifolius*	[66]
Centaureidin	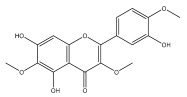	*E. integrifolius*	[66]
Axillarin	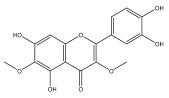	*E. integrifolius*	[66]
Genkwanin	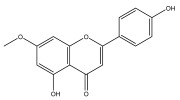	*E. albicaulis*	[47]
Apigenin-7-*O*-(6″-trans-pcoumaroyl- ß -D-glucopyranoside	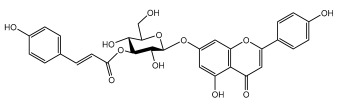	*E. orientalis*	[57]
		*E. spinosus*	[77]
5,7-dihydroxy-8,4′-dimethoxyflavanone-5-*O*-α-L-rhamno-pyranosyl-7-*O*-ß-D-arabinopyranosyl (1→4)-*O*-ß-D-glucopyranoside		*E. echinatus*	[82]
Candidone	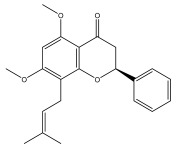	*E. giganteus*	[32]
Chlorogenic acid	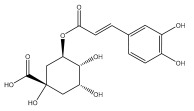	*E. grijisii*	[36]
Cynarin	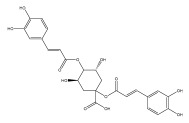	*E. grijisii*	[36]
Rutin	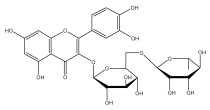	*E. heterophyllus*	[81]
		*E. albicaulis*	[47]
(+)-4-(3-methylbutanoyl)-2,6-di(3,4-dimethoxy)phenyl-3,7-dioxabicyclo[3.3.0]octane	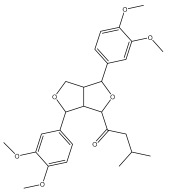	*E. giganteus*	[65]
(+)-4-hydroxy-2,6- di(3,4-dimethoxy)phenyl-3,7-dioxabicyclo[3.3.0]octane	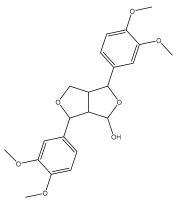	*E. giganteus*	[65]
		*E. giganteus*	[31]
		*E. giganteus*	[32]
Jaceidin (5,7-dihydroxy-2-(4-hydroxy-3-methoxyphenyl)-3,6-dimethoxychromen-4-one)	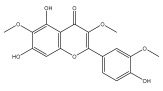	*E. integrifolius*	[66]
centaureidin (5,7,3′-Trihydroxy-3,6,4′-trimethoxyflavone)	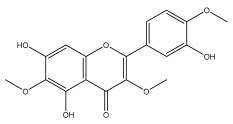	*E. integrifolius*	[66]
Hispidulin (4′,5,7-Trihydroxy-6-methoxyflavone)	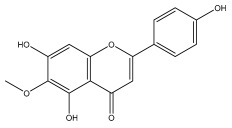	*E. integrifolius*	[66]
axillarin	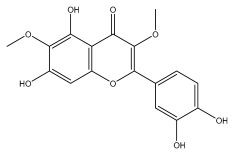	*E. integrifolius*	[66]
Hexacosyl-(*E*)-ferulate	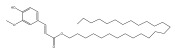	*E. nanus*	[41]
Umbelliferone	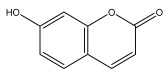	*E*. *integrifolius*	[66]
Syringin	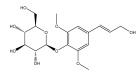	*E. grijisii*	[36]
1,5-dicaffeoylquinic acid	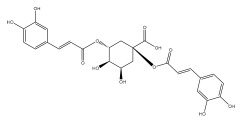	*E. galalensis*	[71]
		*E. ritro*	[83]
3,5-dicaffeoylquinic acid (isochlorogenic acid A)	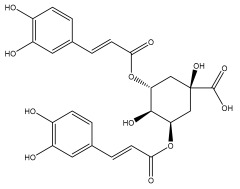	*E. ritro*	[83]
3,4-dicaffeoylquinic acid (isochlorogenic acid C)	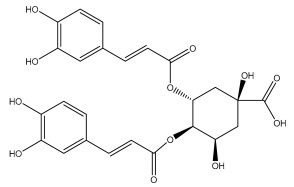	*E. ritro*	[53]
4,5-dicaffeoylquinic acid	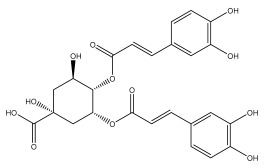	*E. ritro*	[53]

## Data Availability

Not applicable.
